# Molecular-cytogenetic analysis of diploid wheatgrass *Thinopyrum bessarabicum* (Savul. and Rayss) A. Löve

**DOI:** 10.3897/CompCytogen.v13i4.36879

**Published:** 2019-12-03

**Authors:** Ekaterina D. Badaeva, Sergei A. Surzhikov, Alexander V. Agafonov

**Affiliations:** 1 N.I. Vavilov Institute of General Genetics, Russian Academy of Sciences. Gubkina str. 3, Moscow 117333, Russia Engelhardt Institute of Molecular Biology, Russian Academy of Sciences Moscow Russia; 2 Engelhardt Institute of Molecular Biology, Russian Academy of Sciences. Vavilova str. 34, Moscow 117334, Russia N.I. Vavilov Institute of General Genetics, Russian Academy of Sciences Moscow Russia; 3 Central Siberian Botanical Garden, Russian Academy of Sciences, Siberian Branch, Zolotodolinskaya st., 101, Novosibirsk 630090, Russia Central Siberian Botanical Garden, Russian Academy of Sciences Novosibirsk Russia

**Keywords:** Chromosome, evolution, *FISH*-karyotyping, J genome, rRNA gene distribution, *Thinopyrum bessarabicum*

## Abstract

*Thinopyrum bessarabicum* (T. Săvulescu & T. Rayss, 1923) A. Löve, 1980 is diploid (2n=2x=14, JJ or E^b^E^b^), perennial self-fertilizing rhizomatous maritime beach grass, which is phylogenetically close to another diploid wheatgrass species, *Agropyron elongatum* (N. Host, 1797) P. de Beauvois, 1812. The detailed karyotype of *Th. bessarabicum* was constructed based on FISH with six DNA probes representing 5S and 45S rRNA gene families and four tandem repeats. We found that the combination of pAesp_SAT86 (= pTa-713) probe with pSc119.2 or pAs1/ pTa-535 allows the precise identification of all J-genome chromosomes. Comparison of our data with the results of other authors showed that karyotypically *Th. bessarabicum* is distinct from *A. elongatum*. On the other hand, differences between the J-genome chromosomes of *Th. bessarabicum* and the chromosomes of hexaploid *Th. intermedium* (N. Host, 1797) M. Barkworth & D.R. Dewey, 1985 and decaploid *Th. ponticum* (J. Podpěra, 1902) Z.–W. Liu & R.–C. Wang, 1993 in the distribution of rDNA loci and hybridization patterns of pSc119.2 and pAs1 probes could be an indicative of (1) this diploid species was probably not involved in the origin of these polyploids or (2) it could has contributed the J-genome to *Th. intermedium* and *Th. ponticum*, but it was substantially modified over the course of speciation

## Introduction

*Thinopyrum bessarabicum* (T. Săvulescu & T. Rayss, 1923) A. Löve 1980 (syn. *Agropyron bessarabicum* T. Săvulescu & T. Rayss, 1923 or *A. junceum* (K. Linnaeus, 1753) P. de Beauvois, 1812) is a diploid (2n = 2x = 14, JJ or E^b^E^b^), perennial self-fertilizing rhizomatous maritime beach grass (Dewey 1984; Wang 2011). Phylogenetically it is closely related to another diploid wheatgrass species, *A. elongatum* (N. Host, 1797) P. de Beauvois 1812 (2n = 2x = 14, EE or J^e^J^e^), and in some taxonomical systems they are assigned to a common genomic group (Dvořák 1981; Dewey 1984; Wang and Lu 2014). Other authors, however, showed that genomes of these species are genetically distinct (Wang 1985; Jauhar 1988; Forster and Miller 1989; Moustakas 1991; Linc et al. 2017) and differ from each other in a number of species-specific chromosome rearrangements (Gaál et al. 2018; Grewal et al. 2018). *Th. bessarabicum* is thought to be the parental form of many polyploidy *Thinopyrum* Á. Löve, 1980 species including tetraploid *Th. distichum* (C.P. Thunberg, 1794) Á. Löve 1980, *Th. sartorii* (P.E. Boissier & T. von Heldreich, 1859) Á. Löve 1980 and *Th. junceiforme* (Á. Löve & D. Löve, 1948) Á. Löve 1980 with the genome constitution JJEE or E^b^E^b^E^e^E^e^, and hexaploid *Th. intermedium* (N. Host, 1797) M. Barkworth and D.R. Dewey 1985 (2n = 6x = 42, EEE^st^E^st^StSt) and *Th. junceum* (K. Linnaeus, 1753) Á. Löve 1980 (syn. *Elymus farctus* (D. Viviani, 1808) Runemark ex Melderis 1978) with the genome constitution 2n = 6x = 42, JJJJEE or E^b^E^b^E^b^E^b^E^e^E^e^ (Dewey 1984; Charpentier 1992; Liu and Wang 1993; Chen et al. 1998; Tang et al. 2000; Wang et al. 2010; Wang 2011; Kruppa and Molnar-Lang 2016). Genomes related to the J-genome of *Th. bessarabicum* could also present in decaploid *Th. ponticum* (J. Podpěra, 1902) Z.-W. Liu and R.-C. Wang 1993 (2n = 10x = 70, EEEEEEE^st^E^st^E^st^E^st^ or EEEEEEStStStSt (Chen et al. 1998).

The natural distribution range of *Th. bessarabicum* spans along Black sea shore from southeastern and eastern Europe to Turkey (Wang 2011). Because of high tolerance to soil salinity (Gorham et al. 1985; Forster et al. 1987; King et al. 1997; Ceoloni et al. 2015) and pest resistance (Zhang et al. 2002; Xu et al. 2009; Zheng et al. 2014; Grewal et al. 2018), this species is considered as valuable source of useful genes for wheat improvement (William and Mujeeb-Kazi 1993). A number of common wheat-*Th. bessarabicum* amphiploids, disomic addition, substitution, and recombinant lines were produced and characterized using molecular, genetic and cytogenetic methods (William and Mujeeb-Kazi 1993; Zhang et al. 2002; Qi et al. 2010; Patokar et al. 2016; Du et al. 2017; Grewal et al. 2018; Hamdani et al. 2018). As a result of analysis of wheat-*Th. bessarabicum* recombinant lines using a combination of cytogenetic technique with high-throughput genotyping, the homoeologous relationships of all individual *Th. bessarabicum* chromosomes with common wheat chromosomes were established (Grewal et al. 2018). A significant syntenic relationship between the seven linkage groups of *Th. bessarabicum* and their orthologous chromosomes from A, B and D genomes of *Triticum aestivum* K. Linnaeus, 1753 was shown. As a diploid wheat, *Th. bessarabicum* carries a species-specific translocation between 4J and 5J chromosomes, but it possesses additional centomeric translocation between 2J and 5J and a paracentric inversion of 7JS chromosome (Grewal et al. 2018).

*Th. bessarabicum* is characterized by symmetric karyotype consisting of metacentric and submetacentric chromosomes. Four chromosomes carry satellites (SAT) on their short arms. Due to similarity of size and morphological parameters of the J-genome chromosomes, additional methods are necessary for their identification.

The C-banding technique, which was broadly used at the end of XX^th^ for chromosome identification in wheat and related species, was also employed for the analysis of *Th. bessarabicum* chromosomes (Endo and Gill 1984; William and Mujeeb-Kazi 1993; Mirzaghaderi et al. 2010). These studies showed that the J-genome chromosomes possess Giemsa C-bands in subtelomeric regions of either one or both chromosome arms, and small intercalary heterochromatin blocks appear in perinucleolar regions of the SAT chromosomes (Endo and Gill 1984; William and Mujeeb-Kazi 1993). The lack of diagnostic intercalary C-bands restricts applicability of this method for *Th. bessarabicum* chromosome identification.

Fluorescence *in situ* hybridization or FISH provides a broad prospective for plant chromosome analysis. This approach has already been applied for *Th. bessarabicum*, and a standard set of probes – 45S rDNA, pSc119.2, or pAs1 was used for chromosome identification (Du et al. 2017; Linc et al. 2017; Grewal et al. 2018). Besides them, Du et al. (2017) developed several novel J-genome specific oligo-probes with predominantly subtelomeric location for the detection of alien chromatin in wheat-*Th. bessarabicum* introgression lines.

In a current study we mapped six “classical” DNA probes, including 45S and 5S rDNAs (Gerlach and Bedbrook 1979, Gerlach and Dyer 1980), pSc119.2 (Bedbrook et al. 1980), pAs1 (Rayburn and Gill 1986) together with two recently isolated DNA sequences pTa-535 (Komuro et al. 2013) and pAesp_SAT86 (Badaeva et al. 2015) on chromosomes of diploid *Th. bessarabicum* to develop molecular karyotype of this species. Two polyploid *Thinopyrum* species – *Th. intermedium* and *Th. ponticum*, which presumably contain the J-genome, were included in the investigation in order to verify the relationships between species.

## Material and methods

*Thinopyrum* accessions used in analyses, their origin and genome constitution are given in Table 1.

Fixation of the material, slide preparation and fluorescence *in situ* hybridization (FISH) were carried out as described earlier (Badaeva et al. 2017). The oligo-probes pSc119.2, pAs1-1, and pTa-535-1 labelled at the 5’ end with fluorescein (pSc119.2, pAs1) or with Cy-3 (pAs1 and pTa-535) were synthesized in the Laboratory of Biological Microchips of the Engelhardt Institute of Molecular Biology RAS (Moscow, Russia) according to Tang et al. (2014). The probes pTa71, pTa794, and pAesp_SAT86 were prepared by labeling plasmid DNA with fluorescein-12 dUTP or biotin-16-dUTP (Roche, Germany) using nick-translation kit (Roche, Germany). The slides were analyzed on a Zeiss Imager D1 microscope. Metaphase plates were photographed at magnification 100× with a black and white digital camera Axiocam HRm using a software AxioVision, release 4.6. The images were processed using Adobe Photoshop, version 7.0.

**Table 1.** List of materials studied and their origin.

No	Species	Accession #	2*n*	Ploidy level	Genome composition (per 1*n*)*	Origin	Donor name
1	*Thinopyrum bessarabicum*	W6 10232	14	2×	J or E^b^	Russia, Crimea	USDA-ARS (U.S.A.)
2	*Th. bessarabicum*	PI 531711	14	2×	J or E^b^	Russia, Crimea	USDA-ARS (U.S.A.)
3	*Th. intermedium*	–	42	6×	E^st^E^st^St or EtEst(V-J-R)	Russia, unknown	obtained from collection of Moscow Scientific-Research Agricultural Institute of Nonchernozem Zone “Nemchinovka”
4	*Th. ponticum*	–	70	10×	EEEE^st^E^st^ or EEEStSt	Russia, on a sea shore of the island Sergeevskyi, White sea	collected by Dr. A.A. Pomortsev, Vavilov Institute of General Genetics RAS, Moscow, Russia

* – Genome symbols are given according to Wang (2011).

## Results

**Figure 1.**
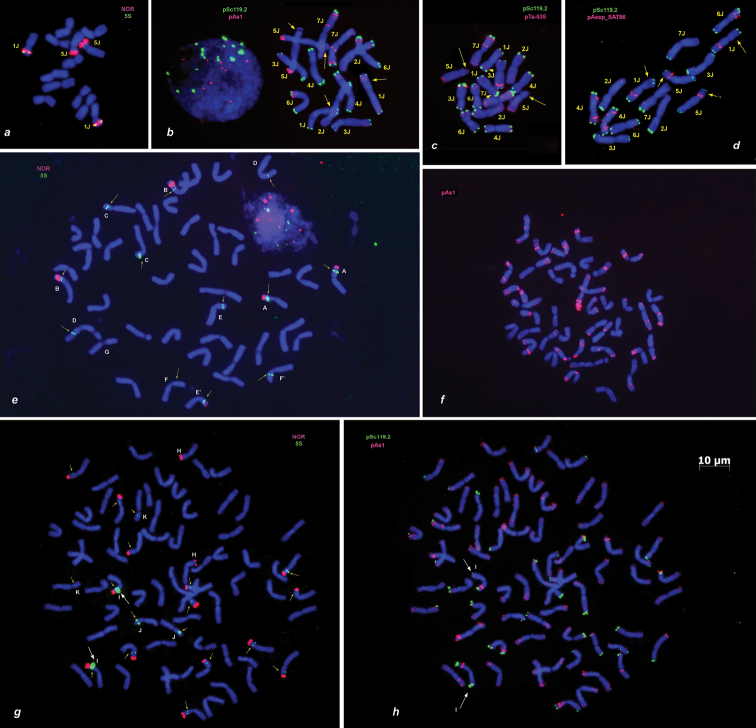
Distribution of rDNA probes and different tandem repeats on metaphase chromosomes of perennial grass species: *Th. bessarabicum* W6 10232 (**a–c**) and PI 531711 (**d**), *Th. intermedium* (**e, f**); and *Th. ponticum* (**g, h**). Probe combination in **a, e, g** pTa71, red + pTa794, green **b, h** pSc119.2, green + pAs1, red **c** pSc119.2, green + pTa-535, red **d** pSc119.2, green + pAesp_SAT86, red **f** pAs1, red. The letters from A to K designate pairs of homologous chromosomes identified in *Th. intermedium* (**e**) or *Th. ponticum* (**g**) mitotic cells based on characteristic patterns of 5S and/or 45S rDNA probes. Yellow arrows (**b–d**) show position of secondary constrictions on *Th. bessarabicum* chromosomes. 5S rDNA sites on *Th. intermedium* (**e**) or *Th. ponticum* (**g**) chromosomes are indicated with small arrows. White arrows (**h**) show homologous *Th. ponticum* chromosomes with contrasting pSc119.2 patterns. Scale bar: 10 µm.

FISH with pTa71 probe revealed four prominent 45S rDNA signals in the regions of secondary constrictions of two pairs of *Th. bessarabicum* chromosomes (Fig. 1a). Two large pTa794 (5S rDNA) sites were found on a chromosome pair carrying large satellites. They were located on satellites, distally to NORs, which is typical for the genetic group 1 of the Triticeae. Very tiny 5S rDNA signals appeared occasionally in the middle of short arm of the second pair of SAT chromosomes. As far as signals were observed in some, but not all cells, they were not considered in the analysis.

**Figure 2.**
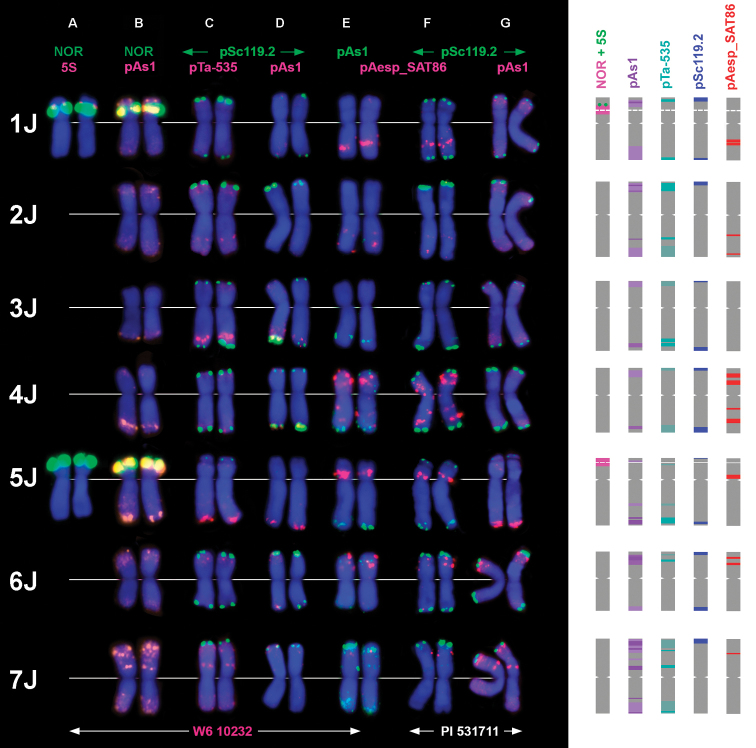
Distribution of different tandem repeats on *Th. bessarabicum* chromosomes; their idiograms are given on the right. The probe combinations are shown on the top, probe color corresponds to signal color. 1 – 7 – genetic groups. The pAs1 probe on lanes B, and G was labelled with Cy-3/TAMRA, while on lanes D and E with fluorescein resulting in lower pAs-1 signal intensities.

Hybridization pattern of oligo-pAs1 and oligo-pSc119.2 probes obtained in a current study (Fig. 1b) corresponded to those published earlier by Grewal et al. (2018), which allowed us to classify the J-genome chromosomes according to genetic nomenclature reported in this paper. Unequal pSc119.2-sites were present in subterminal regions of either both (1J, 3J, 4J, 6J) or only one chromosome arm (2JS, 5JS, 7JS). The largest pSc119.2 signals were observed on 2JS, 4J, and 6J, whereas chromosome 5J had the smallest signals (Figs 1b–d, 2).

Hybridization with pAs1 probe resulted in fuzzy labelling of distal chromosome halves; signal intensities varied from medium to relatively high depending on a chromosome and fluorochrome used (signals generated by Fluorescein-labelled pAs1 probe (Fig. 2, lanes D, E) were always weaker than signals of the same probe labelled with Cy3 or TAMRA (Fig. 2, lanes B, G), and only strongest FITC-signals were visualized by FISH). Most intense pAs1-signals were found on 5JL, 6JS, and in the distal and median regions of the 7J short arm (Figs 1b, 2). Labelling patterns of pTa-535 probe (Figs 1c, 2) were similar to those of pAs1, although pTa-535 signals on 3JL were significantly stronger, while those on 1J – slightly weaker compared to pAs1.

Hybridization with the pAesp_SAT86 probe produced sharp, large diagnostic signals on four (1J, 4J, 5J, and 6J) out of seven pairs of *Th. bessarabicum* chromosomes (Figs 1d, 2). Labelling patterns were identical in both *Th. bessarabicum* accessions and, in combination with either pSc119.2 or pAs1/ pTa-535, allowed the precise identification of all J-genome chromosomes. The chromosome 1J was characterized by bright double signals in the middle of long arm, and 5J contained diagnostic prominent signal in the short arm, adjacent to the centromere (Figs 1d, 2). Chromosomes 4J and 6J, which were hardly distinguishable based on pSc119.2 and pAs1 labelling patterns, were easily discriminated using the pAesp_SAT86 probe. Chromosome 4J carried two prominent signals in the short and two in the long arm, while the chromosome 6J was characterized by double pAesp_SAT86 sites in a distal part of the short arm (Figs 1d, 2).

Relatively faint pAesp_SAT86 signals were detected on chromosomes 2J and 7J, which both carried sharp pSc119.2 sites in their short arms (Fig. 2). A single pAesp_SAT86 signal was found on the short arm of 7J (containing pSc119.2 site), whereas two wheak signals appeared on the long arm of 2J (lacking pSc119.2 site). No pAesp_SAT86 hybridization sites were detected on the chromosome 3J.

FISH with pTa71 and pTa794 probes on hexaploid *Th. intermedium* revealed twelve 5S rDNA signals (Fig. 1e, arrowed), five of which were co-localized with NORs (chromosomes A, B, E/E’), which were found in subterminal regions of the same chromosome arms. The remaining 5S sites were distributed among seven other chromosomes (designated C-F on Fig. 1e) in either distal or proximal position of the arm. In addition, a weak 45S rDNA signal was detected approximately in the middle of short arm of a single chromosome designated G. Such asymmetric distribution of rDNA clusters among *Th. intermedium* chromosomes can be the consequence of unbalanced translocations that could have occurred in the genome of this perennial, vegetatively propagated plant and then maintained in a progeny over years or even decades. High number of unbalanced translocations was also detected by FISH with pAs1 probe in another *Th. intermedium* genotype (Fig. 1f): at least eighteen out of 21 homologous chromosomes pairs exhibited different labeling patterns, which significantly complicated their identification.

Eighteen chromosomes of decaploid *Th. ponticum* possessed 5S rDNA clusters of variable sizes (Fig. 1g, indicated with small arrows), fourteen of them also carried terminal NORs. Only one chromosome pair designated I, can be distinguished from others based on the extremely large pTa794 (5S rDNA) signals. Two different chromosome pairs lacking NORs contained 5S rDNA loci significantly different in size (chromosomes J and K, Fig. 1g), while another chromosome pair – H, possesses only terminal large 45S rDNA signals, like the chromosome 5J of *Th. bessarabicum*. Subsequent hybridization of pSc119.2 and pAs1 probes on the same metaphase cell revealed distinct pSc119.2 sites in subtelomeric regions of one or both arms nearly in a half of *Th. ponticum* chromosomes (Fig. 1h). Polymorphism of hybridization patterns was observed between homologous chromosomes (Fig. 1h, chr. I, shown with white arrows). The pAs1 signals were located in distal regions of nearly all chromosomes, however, owing to high ploidy level, similar location and high polymorphism, pAs1-labelling patterns did not allow identification of all *Th. ponticum* chromosomes.

## Discussion

Diploid *Th. bessarabicum* is considered as one of genome donors to *Th. intermedium* (Chen et al. 1998; Liu and Wang 1993; Wang et al. 2010) and *Th. ponticum* (Chen et al. 1998). The molecular karyotype of intermediate wheatgrass has been recently constructed by Cui et al. (2018) and Yu et al. (2019) based on tandemly repeated DNA. In addition, the 5S and 45S rDNA probes were mapped on chromosomes of several *Th. intermedium* genotypes by Mahelka et al. (2013) and Yu et al. (2019). Molecular karyotypes were developed for other diploid and polyploid wheatgrass species (Brasileiro-Vidal et al. 2003; Linc et al. 2012, 2017; Li et al. 2016a, b, 2018; Said et al. 2018), thus permitting their comparison to assess genome relationships.

The distribution of rDNA loci is often used in phylogenetic studies of plants. In the Triticinae, major NORs can be located on group 1, 5 and 6 chromosomes (Appels et al. 1980), whereas the 5S rDNA loci appear on group 1 and 5 chromosomes (Dvořák et al. 1989). The number and relative position of 45S and 5S rDNA clusters on chromosomes of diploid *Aegilops* K. Linnaeus, 1753 (Badaeva et al. 1996) or *Hordeum*, K. Linnaeus, 1753, species (Taketa et al. 2001) is found to be highly diverse, but conservative for each genomic group. Genome-specific patterns of rRNA gene probes were also reported for several diploid wheatgrass species – *Agropyron elongatum*, *A. cristatum* (K. Linnaeus, 1753) J. Gaertner 1770, *Th. bessarabicum*, *Dasypyrum villosum* (K. Linnaeus, 1753) T. Candargy 1901 and *D. breviaristatum* (H. Lindberg, 1932) Frederiksen 1991, with genome constitutions E, P, J, V and V^b^ respectively (Dvořák et al. 1984; Liu et al. 2010; Linc et al. 2012, 2017; Zhang et al. 2013; Li et al. 2016a, 2018; Said et al. 2018).

Earlier Linc et al. (2017) revealed two pairs of major NORs in karyotypes of the three diploid wheatgrass species, *Th. bessarabicum*, *A. elongatum*, and *Pseudoroegneria spicata* (F.T. Pursh, 1813) Á. Löve 1980. The SAT chromosomes of *Th. bessarabicum* were assigned to homoeologous groups 5 and 6 by analogy with *A. elongatum*, which carries NORs on chromosomes 5E and 6E (Dvořák et al. 1984; Linc et al. 2012; Li et al. 2018). Based on relative position of 5S and 45S rDNA loci and taking into consideration the similarity of pAs1 and pSc119.2-labelling patterns with chromosomes 1J and 5J reported by Grewal et al. (2018), we concluded that the SAT chromosomes of *Th. bessarabicum* belong to genetic groups 1 and 5.

Both *Th. bessarabicum* and *A. elongatum* contain a pair of 5S rDNA loci on group 1 chromosomes. Major clusters of 45S rDNA probe are located on group 1 and 5 chromosomes of *Th. bessarabicum* (Grewal et al. 2018), but on chromosomes 5E and 6E of *A. elongatum* (Dvořák et al. 1984; Linc et al. 2012; Li et al. 2018), which contains additional minor NORs on 1ES (Li et al. 2018). Based on dissimilarity of rDNA probe distribution we conclude that the J-genome of *Th. bessarabicum* is genetically distinct from the E-genome of *A. elongatum*.

Interestingly, polyploid *Thinopyrum* possess higher number of 5S rDNA loci per 1x compared to diploids species. Thus, we detected twelve pTa794 sites (two per 1x) in hexaploid *Th. intermedium* (Fig. 1e, indicated with small arrows), five of them were co-localized with NORs. From nine to ten 5S rDNA signals (1.5–1.67 per 1x) were revealed in four *Th. intermedium* genotypes by Mahelka et al. (2013). Yu et al. (2019) found twelve 5S and six 45 rDNA loci in intermediate wheatgrass; two chromosome pairs from the J-genome and one pair from St genome showed hybridization sites of both probes. In all cases the chromosomes carrying clusters of both rDNA families, displayed an identical signal arrangement: the 5S rDNA site was always located proximally to NOR.

We found similar pattern in decaploid *Th. ponticum* (Fig. 1f). Earlier Brasileiro-Vidal et al. (2003) reported that 17 chromosomes of *Th. ponticum* possessed both 45S and 5S rDNA sites, and the 5S rDNA sites were located proximally to NORs. Li and Zhang (2002) suggested that exclusively terminal position of 45S rDNA clusters is a secondary trait that has emerged during evolution of polyploid species. However, such arrangement of ribosomal probes was found only in diploid wheats (Dubcovsky and Dvořák 1995, Badaeva et al. 2015), but it was not observed in *Aegilops* (Badaeva et al. 1996), or the J-genome of *Th. bessarabicum* (Fig. 1a). Therefore, *Th. bessarabicum* was probably not involved in the origin of these polyploids or the J-genome was significantly modified during speciation.

The karyotype of *Th. bessarabicum* shared many common features with karyotypes of other diploid grasses. These are distinct pSc119.2 sites in subtelomeric chromosome regions and high amount of pAs1 repeat, which is accumulated predominantly in the distal chromosome halves (Zhang et al. 2013; Li et al. 2016a, 2018; Du et al. 2017; Linc et al. 2017; Grewal et al. 2018; Said et al. 2018). This or related repeats belonging to the same *Afa*-family are highly abundant in the D-genome of *Aegilops tauschii* Cosson, 1850, in the A-genome of diploid wheat (Megyeri et al. 2012), the I-genome *Hordeum* species (Taketa et al. 2000), diploid and polyploid species from *Elymus* K. Linnaeus, 1753, *Leymus* C.F.F. Hochstetter, 1848, and *Psathyrostachys* S.A. Nevsky, 1933 genera (Nagaki et al. 1999; Dvořák 2009). *Th. bessarabicum* is similar to *Ae. tauschii* and diploid wheat also in a high amount of pTa-535 repeat, which is detected in genomes of *D. breviaristatum* (Li et al. 2016a), *Th. elongatum* (Li et al. 2018) and in the J and J^s^ genomes of intermediate wheatgrass (Yu et al. 2019).

As was shown in a current study, the sequence pAesp_SAT86 (= pTa-713) hybridizes specifically to six out of seven *Th. bessarabicum* chromosomes. Probe distribution is species-specific, because it differs from the pTa-713 labeling patterns of wheat (Komuro et al. 2013; Badaeva et al. 2015), *Aegilops* (Ruban and Badaeva 2018) or *A. elongatum* (Li et al. 2018) chromosomes. The pTa-713 signals are detected on chromosomes 1E, 4E, 5E and 7E of *A. elongatum* (Li et al. 2018). Orthologous chromosomes of *Th. bessarabicum* and *A. elongatum* belonging to group 4 and 5 display similar, while of other groups – different patterns. This can be due to site-specific sequence amplification/ elimination or species-specific chromosomal rearrangements identified in both species (Gaál et al. 2018; Grewal et al. 2018), which further confirms the distinctness of their genomes.

## Conclusion

A detailed karyotype of *Th. bessarabicum* was constructed using FISH with six DNA probes representing 5S and 45S rDNAs and four tandem repeats belonging to different families. A combination of pAesp_SAT86 (= pTa-713) probe with either pSc119.2 or pAs1/ pTa-535 was found to be most effective for the identification of J-genome chromosomes. Comparison of our results with data available from literature showed that the J-genome of *Th. bessarabicum* is distinct from genomes of other diploid wheatgass species. Differences between chromosomes of *Th. bessarabitum*, on one hand, and *Th. intermedium* and *Th. ponticum*, on the other hand, indicate that probably *Th. bessarabitum* did not contribute genome to these polyploid species. Alternatively, the J-genome could be present in polyploid wheatgrasses, but in significantly rearranged form.

All authors declare that there is no conflict of interests exists. All of the authors have contributed substantially to the manuscript and approved the submission.
